# *In Vitro* Activity of Peptide Antibiotics in Combination With Other Antimicrobials on Extensively Drug-Resistant *Acinetobacter baumannii* in the Planktonic and Biofilm Cell

**DOI:** 10.3389/fphar.2022.890955

**Published:** 2022-05-11

**Authors:** Qianlin Meng, Fei Lin, Baodong Ling

**Affiliations:** ^1^ Sichuan Province College Key Laboratory of Structure-Specific Small Molecule Drugs, Chengdu Medical College, Chengdu, China; ^2^ School of Pharmacy, Chengdu Medical College, Chengdu, China; ^3^ Department of Pharmacy, The First Affiliated Hospital of Chengdu Medical College, Chengdu, China

**Keywords:** peptide antibiotics, antimicrobials, *Acinetobacter baumannii*, synergy, antibacterial

## Abstract

*Acinetobacter baumannii* is one of the most dangerous opportunistic pathogens in the global health care setup. Its drug resistance and biofilm-forming capability are often associated with chronic infections that are difficult to treat. Therefore, the clinical treatments for highly drug-resistant *A. baumannii* are limited. Antimicrobial peptides are broad-spectrum antibacterial agents combined with antibiotics that minimize selective bacterial resistance and enhance antibacterial efficacy. The current study evaluated the synergistic antibacterial activities of clinically important peptide antibiotics combined with other antimicrobials against nine extensively drug-resistant *A. baumannii* strains in planktonic and biofilm cells *in vitro*. Polymyxin B and E combined with imipenem showed 100% synergy in the planktonic cell with the checkerboard. Moreover, polymyxin E with rifampicin and bacitracin with imipenem or meropenem showed 100% additive effects. In the biofilm cell, polymyxin B and E combined with azithromycin showed 100% synergy, when vancomycin with azithromycin, rifampicin, and bacitracin with azithromycin or rifampicin, and teicoplanin with tigecycline or rifampicin, all showed 100% additive effects. Therefore, peptide antibiotics combined with other antimicrobials have synergistic or additive effects on extensively drug-resistant *A. baumannii* in planktonic and biofilm cells. In addition, the combination of polymyxins with carbapenems or azithromycin could be an ideal therapy against extensively drug-resistant *A. baumannii* infections.

## Introduction

*Acinetobacter baumannii*, an opportunistic nosocomial pathogen causing severe infections, poses a significant threat to worldwide public health (Lee et al., 2017). The World Health Organization has categorized carbapenem-resistant *A. baumannii*, a primary member of the ESKAPE, as one of the first priority resistant bacteria globally (Mulani et al., 2019; De Oliveira et al., 2020). *A. baumannii* possess intrinsic antimicrobial resistance with its ability to easily adopt new resistance mechanisms that has driven the evolution of extensive-drug and even pan-drug-resistance. (Sobouti et al., 2020). *A. baumannii* mainly exists in two forms in the environment: planktonic and biofilm cells (Koo et al., 2017). Extensively drug-resistant *A. baumannii* (XDRAB) has acquired drug resistance features and strong biofilm-forming ability, a significant source of persistent nosocomial infection (Mohammed et al., 2020). Due to the lack of antibacterial drugs against XDRAB, new combinations are urgently required to replace traditional antibacterial therapy, which has become the current international research hotspot in the healthcare sector (Tacconelli et al., 2018).

Antimicrobial peptides (AMPs) both natural and synthetic are one of the primary options for overcoming drug resistance (Sierra et al., 2017). Moreover, some peptides, especially those that are similar to animal defense antimicrobial peptides, have immunomodulatory activity (Jenssen et al., 2006), which have less bacterial resistance and low cytotoxicity in humans (Pletzer and Hancock, 2016). AMPs in combination with antibiotics have been shown to minimize selective bacterial resistance and enhance antibacterial efficacy (Jahangiri et al., 2021). Currently, new AMPs still require large-scale clinical trials for validation (Liu et al., 2016). Therefore, re-evaluating the existing clinical peptide antibiotics through non-traditional combinations could be a significant opportunity against XDRAB.

In this study, we investigated the antibacterial and synergistic effects of peptide antibiotics (e.g., polymyxin B, polymyxin E, vancomycin, bacitracin, and teicoplanin) in combination with other antimicrobials (e.g., imipenem, meropenem, azithromycin, and rifampicin) against XDRAB in the planktonic and biofilm cells, that could provide a new therapy for preventing and controlling XDRAB infection*.*

## Materials and Methods

### Drugs and Reagents

Clinically-used antibacterial agents (Magiorakos et al., 2012), such as piperacillin, ceftazidime, cefotaxime, tetracycline, minocycline, gentamicin, amikacin, ciprofloxacin, levofloxacin, chloramphenicol, imipenem, meropenem, tigecycline, polymyxin B, polymyxin E, vancomycin, bacitracin, and teicoplanin were purchased from Meilun Biological (Dalian, Liaoning, China). In addition, cation-adjusted MH broth (CAMHB), Muller-Hinton broth (MHB) and tryptic soy broth (TSB) medium were obtained from Haibo Biotechnology (Qingdao, Shandong, China).

### Bacterial Strains

Nine XDRAB strains were isolated from clinical specimens such as the septum, urine, or respiratory lavage fluid of patients from the respiratory intensive care unit, intensive care unit, the cardiothoracic surgery department, and the neurology department at the First Affiliated Hospital of Chengdu Medical College during the period from 2018 to 2020 (Chengdu, Sichuan, China). In addition, *A. baumannii* ATCC 19606, *Staphylococcus aureus* ATCC 29213, and *Escherichia coli* ATCC 25922 were used as quality control strains in antimicrobial susceptibility testing (AST) and obtained from the American Type Culture Collection (ATCC, Manassas, VA, United States).

### Antimicrobial Susceptibility Test

The minimum inhibitory concentrations (MICs) were determined by the broth microdilution method for the antibacterial agents and peptide antibiotics for *A. baumannii* based on the guidelines provided by the Clinical and Laboratory Standards Institute (CLSI) (Humphries et al., 2021). Briefly, bacterial cells were inoculated in Luria–Bertani agar medium at 37°C for 16–20 h, and then resuspended in saline (0.9% sodium chloride) to adjust the bacterial content to reach the 0.5 McFarland turbidity standard, followed by a 20-fold dilution. Antimicrobial solutions were prepared according to the CLSI (Humphries et al., 2021). Finally, 180 μl CAMHB, 10 µl corresponding antimicrobials, and 10 µl bacterial resuspension were added into 96-well plate (NEST TC, 701001, www.cell-nest.com). The value of OD_600_ was measured after incubating at 37°C for 16–20 h.

### Biofilm Susceptibility Test

The minimum biofilm inhibitory concentrations (MBICs) were detected using a previous study protocol (Kart et al., 2021) for the antibacterial agents and peptide antibiotics for *A. baumannii* in the biofilm cell. First, bacterial cells were inoculated in Luria–Bertani agar medium at 37°C for 16–20 h. Next, the bacterial monoclones were resuspended in saline (0.9% sodium chloride) to adjust the bacterial content to reach the 0.5 McFarland turbidity standard. Then, 190 µl TSB and 10 µl bacterial resuspension were mixed in a 96-well plate. After incubating at 37°C for 24 h, the medium was aspirated, and 200 µl PBS was added to wash three times. Finally, 190 μl MH broth medium and 10 µl corresponding antibiotics were added into 96-well plate (NEST TC, 701001, www.cell-nest.com). The optical density was measured at OD_600_ after incubating for 16–20 h at 37°C.

### Synergistic Studies in the Planktonic and Biofilm Cell

The checkerboard method, the fractional inhibitory concentration index (FICI), and the fractional biofilm inhibitory concentration index (FBIC) evaluated the synergistic effects of five peptide antibiotics combined with one of the five antimicrobials in the planktonic and biofilm cells among the nine XDRAB isolates. The planktonic cells were investigated by adding 170 µl of MHB and 10 µl of bacterial suspension (1.5 × 10^8^ CFU/ml) into the 96 well plates. It was followed by adding 10 µl of serially diluted peptide antibiotics along the x-axis and antimicrobials along the y-axis. The biofilm cells were studied by cultivating the bacteria to form the biofilm into a 96-well plate (NEST TC, 701001, www.cell-nest.com) and washed with 200 µl of PBS three times. The following steps were consistent with the planktonic study. The value of OD_600_ was measured after incubating at 37°C for 20 h. The FICI and FBIC were evaluated as follows: Synergy between the two drugs was defined as either a FICI or an FBIC of ≤0.5, >0.5–1 for additivity, >1–2 for indifference, and >2 for antagonism (Kim et al., 2015; Peng et al., 2020; Lin et al., 2021).

## Results

### Antimicrobial Susceptibility Test in the Planktonic and Biofilm Cell

The MICs and MBICs of 15 antibacterial agents with five peptide antibiotics are shown in Table 1, based on the interpretive standards established by CLSI (Humphries et al., 2021). These isolates from the planktonic cell were either susceptible or intermediate to polymyxin B and polymyxin E. The tigecycline sensitivity rate was 100%, but they were resistant to other antibacterial agents. The drug-resistance profiles defined the isolates as XDRAB (Magiorakos et al., 2012). Once the biofilm of XDRAB was formed, the polymyxin and tigecycline resistance in the biofilm cell was further enhanced, depicting a pan-drug resistance (PDR) trend. In addition, there was no significant difference in the antibacterial effects of the five peptide antibiotics on XDRAB under the planktonic and biofilm cells; only the drug resistance of the latter was further elevated.

**TABLE 1:** Minimum inhibitory concentrations (MICs) and Minimum biofilm inhibitory concentrations (MBICs) of 15 antibacterial agents with five peptide antibiotics against nine clinical isolates of *A. baumannii.*

Antibiotics	MIC/MBIC (μg/ml) for isolates										Resistance phenotype (%)
		AB1	AB2	AB3	AB4	AB5	AB6	AB7	AB8	AB9	
Piperacillin	MIC	>1024	>1024	>1024	>1024	>1024	>1024	>1024	>1024	>1024	R (100%)
	MBIC	>1024	>1024	>1024	>1024	>1024	>1024	>1024	>1024	>1024	R (100%)
Ceftazidime	MIC	256	256	256	256	256	256	256	256	256	R (100%)
	MBIC	1024	1024	1024	1024	1024	1024	512	1024	512	R (100%)
Cefotaxime	MIC	256	256	256	256	256	256	256	256	256	R (100%)
	MBIC	512	1024	1024	512	512	512	512	512	512	R (100%)
Tetracycline	MIC	>1024	>1024	>1024	>1024	>1024	>1024	>1024	>1024	>1024	R (100%)
	MBIC	>1024	>1024	>1024	>1024	>1024	>1024	>1024	>1024	>1024	R (100%)
Minocycline	MIC	16	16	16	16	16	16	16	16	16	R (100%)
	MBIC	16	32	32	16	16	16	16	16	32	R (100%)
Gentamicin	MIC	>1024	>1024	>1024	>1024	>1024	>1024	>1024	>1024	>1024	R (100%)
	MBIC	>1024	>1024	>1024	>1024	>1024	>1024	>1024	>1024	>1024	R (100%)
Amikacin	MIC	>1024	>1024	>1024	>1024	>1024	>1024	>1024	>1024	>1024	R (100%)
	MBIC	>1024	>1024	>1024	>1024	>1024	>1024	>1024	>1024	>1024	R (100%)
Ciprofloxacin	MIC	32	32	32	32	32	32	32	32	32	R (100%)
	MBIC	64	64	64	64	64	128	64	64	64	R (100%)
Levofloxacin	MIC	16	16	16	8	8	16	8	16	16	R (100%)
	MBIC	16	16	16	32	16	32	16	16	32	R (100%)
Chloramphenicol	MIC	32	32	32	32	32	32	32	32	32	R (100%)
	MBIC	256	256	128	256	256	256	128	256	128	R (100%)
Imipenem	MIC	64	64	64	64	64	64	64	64	64	R (100%)
	MBIC	128	128	128	128	64	64	128	128	64	R (100%)
Meropenem	MIC	32	32	32	32	32	32	32	32	32	R (100%)
	MBIC	64	128	128	64	128	128	128	64	64	R (100%)
Tigecycline	MIC	1	1	1	1	1	1	1	1	0.5	S (100%)
	MBIC	2	4	2	4	2	2	2	2	1	S (11%)
											I (67%)
											R (22%)
Polymyxin B	MIC	2	1	1	2	2	1	2	2	2	S (33%)
											I (67%)
	MBIC	16	16	8	8	16	16	8	8	8	R (100%)
Polymyxin E	MIC	2	1	1	2	2	1	2	2	2	S (33%)
											I (67%)
	MBIC	16	8	4	4	8	8	8	4	8	R (100%)
Vancomycin	MIC	64	64	128	64	128	128	128	64	128	R (100%)
	MBIC	256	128	128	128	256	256	128	128	128	R (100%)
Bacitracin	MIC	64	64	128	128	128	128	64	128	128	R (100%)
	MBIC	256	256	256	256	256	256	256	256	256	R (100%)
Teicoplanin	MIC	64	64	32	32	64	32	128	32	64	R (100%)
	MBIC	128	64	64	128	128	64	128	128	64	R (100%)

S = susceptible, I = intermediate, R = resistance.

### Combination Antimicrobial Susceptibility Test in the Planktonic and Biofilm Cell

Based on the FICI and FBIC values obtained from the combined drug susceptibility test (Table 2), five peptide antibiotics combined with other five antimicrobials had different degrees of synergistic or additive effects on nine XDRAB strains in the planktonic and biofilm cells. The FICI range of polymyxin B or polymyxin E combined with imipenem in the planktonic cell was 0.375–0.5 and 0.25–0.5, respectively. Synergy was noted in 100% of the isolates. When polymyxin E was combined with rifampicin and bacitracin with imipenem or meropenem, there was a 100% additive effect. In the biofilm cell, when polymyxin B and polymyxin E were combined with azithromycin, the FBIC range was 0.19–0.31 and 0.19–0.375, respectively, with a 100% synergistic effect. In addition, 100% additivity was detected when vancomycin was combined with azithromycin or rifampicin, bacitracin with azithromycin or rifampicin, and teicoplanin with tigecycline or rifampicin.

**TABLE 2:** Antibacterial effect of 5 peptide antibiotics in combination with 5 antimicrobials on extensively drug-resistant *A. baumannii* isolates (*n* = 9) in the planktonic cell (PC) and biofilm cell (BC).

Antibiotics		FICI range	Interplay (PC)			FBIC range	Interplay (BC)		
			Synergy (%)	Additicity (%)	Indifference (%)		Synergy (%)	Additicity (%)	Indifference (%)
PB	IMP	0.375–0.5	100	0	0	0.31–1	11	89	0
	MER	0.375–0.625	33	67	0	0.5–1	33	67	0
	TIG	0.75–1.5	0	44	56	0.375–0.75	67	33	0
	AZI	0.31–0.625	44	56	0	0.19–0.31	100	0	0
	RIF	0.5–1	11	89	0	0.375–0.625	33	67	0
PE	IMP	0.25–0.5	100	0	0	0.375–0.75	11	89	0
	MER	0.5–0.75	78	22	0	0.25–1	44	56	0
	TIG	0.75–1.5	0	44	56	0.375–1	67	33	0
	AZI	0.25–0.75	44	56	0	0.19–0.375	100	0	0
	RIF	0.56–1	0	100	0	0.375–0.75	56	44	0
VAN	IMP	0.625–1.125	0	22	78	1.03–1.5	0	0	100
	MER	0.56–1.13	0	11	89	0.625–1.13	0	67	33
	TIG	0.56–1.5	0	78	22	0.375–0.625	44	56	0
	AZI	0.625–1.125	0	78	22	0.75–1	0	100	0
	RIF	0.5–1	11	89	0	0.625–1	0	100	0
BAC	IMP	0.625–1	0	100	0	0.375–1	11	89	0
	MER	0.625–0.75	0	100	0	0.5–1	22	78	0
	TIG	0.5–1	11	89	0	0.5–0.75	33	67	0
	AZI	0.31–1	22	78	0	0.75	0	100	0
	RIF	0.56–1.25	0	33	67	0.625–1	0	100	0
TEL	IMP	0.375–0.75	44	56	0	0.5–1	33	67	0
	MER	0.375–0.75	11	89	0	0.5–0.75	33	67	0
	TIG	0.75–1.125	0	78	22	0.625–1	0	100	0
	AZI	0.56–2	0	44	56	0.375–0.75	22	78	0
	RIF	0.51–1.5	0	89	11	0.625–1	0	100	0

(a%) = nine XDRAB, strains showed a% synergy or additicity or indifference in the combination.

PB = polymyxin B, PE = polymyxin E, VAN = vancomycin, BAC = bacitracin, TEL = teicoplanin, IMP = imipenem, MER = meropenem, TIG = tigecycline, AZI = azithromycin, RIF = rifampicin.

## Discussion

Compared with the planktonic cell, the biofilm cell of *A. baumannii* possesses significant biological characteristics at the transcription levels of associated virulence genes (Selasi et al., 2016). Therefore, we evaluated the antibacterial effects of nine XDRAB strains in the planktonic and biofilm cells. We also investigated the antibacterial and synergistic effects of peptide antibiotics, both alone and in combination with other antimicrobials. Our antimicrobial susceptibility results depicted that all the nine isolates in the planktonic cell were 100% sensitive to tigecycline and 33% sensitive to polymyxins. However, they were resistant to the rest of the 13 drugs. These strains all conformed to the criteria of XDRAB (Magiorakos et al., 2012). Our study indicated that the resistance rate to polymyxins was 100% after the XDRAB biofilm formation, while the intermediate tigecycline rate was 67%. The resistance rate was 22%, and the sensitivity rate was 11%, which was consistent with the results of Shenkutie et al. (2020). Various studies have demonstrated that *A. baumannii* drug-resistance in the biofilm cell was significantly elevated, which could be related to the extracellular polysaccharide matrix formation and efflux pump overexpression in biofilm (Singh et al., 2017). Our findings were consistent with the fact that XDRAB has posed a significant threat in global clinical settings requiring combination therapy. Indeed, the clinical options to treat XDRAB with antibacterial drugs are limited. Therefore, a non-traditional combination treatment has been pursued (Spellberg and Bonomo, 2015). New AMPs are mainly produced by living organisms and defend the host against pathogens, as a part of innate immunity (Wang et al., 2016), which could become potential antibacterial agents against XDRAB infection (Duraisamy et al., 2020). However, new AMPs are still in the process of drug discovery and development. Thus, we targeted drugs with existing clinical peptide antibiotics combined with other antimicrobials to study whether they have synergistic effects on XDRAB in planktonic and biofilm cells.

Our study depicted that the synergistic antibacterial effects of polymyxin B or E combined with imipenem were 100% against XDRAB in the planktonic cell. The optimal concentrations of polymyxin with imipenem are 1 μg/ml and 4 μg/ml, respectively. The imipenem MIC ≤4 μg/ml was thought to be susceptible *in vitro*. Our results were consistent with the study by Leu et al. (2014), which showed that the response of imipenem resistance reversal was dependent on polymyxin concentrations, the combination of imipenem and polymyxin at 1 μg/ml could reverse imipenem resistance in 74.6% of *A. baumannii.* Another study recommended polymyxin combined with imipenem as a treatment regimen for extensively drug-resistant Gram-negative bacteria (Guan et al., 2016). Moreover, our study revealed that the combination of polymyxin E with rifampicin provided a 100% additive effect, wherein polymyxin could have disrupted the integrity of the bacterial outer membrane, leading to the elevated intracellular concentration of rifampicin and enhancing the antibacterial effect (Goldstein, 2014; Khondker and Rheinstädter, 2020). Our results were consistent with the study by Park et al. (2019), which showed that the combination of polymyxin and rifampicin has been used successfully against polymyxin-resistant *A. baumannii* pneumonia. Bacitracin is a peptide antibiotic, it’s parenteral administration is highly restricted due to its nephrotoxicity, which prohibits its systemic therapeutic usage (Holbrook et al., 2021). Therefore, bacitracin is commonly used in combination with polymyxin and neomycin as triple antibiotic ointment for the topical treatment of wound infections (Sierra et al., 2017). When combined with imipenem or meropenem to treat XDRAB in the planktonic cell, it has 100% additive effects because bacitracin hindered bacterial cell wall synthesis by inhibiting lipid carrier dephosphorylation (Mascher et al., 2003). In contrast, carbapenems accelerated cell wall defects by restraining the secretion of mucopeptide synthase in the cell membrane. As a result, it inhibited bacterial cell wall synthesis and enhanced the antibacterial effects.

Our study showed that polymyxin B or E combinations with azithromycin had 100% synergistic effects when XDRAB was in the biofilm cell. As a traditional biofilm inhibitor (Parnham et al., 2014), azithromycin could inhibit the synthesis of extracellular polysaccharides and alginate of *Pseudomonas aeruginosa* at the sub-MIC level. The synthesis indirectly affected the expression of related virulence factors, inhibiting the biofilm formation (Imperi et al., 2014). We observed that the MBIC of azithromycin was 512–1024 μg/ml and was reduced to 128 μg/ml when combined with polymyxins. This synergistic effect is due to the inhibition of biofilm-related virulent gene expression by azithromycin through biofilm destruction of XDRAB using polymyxins. On the other hand, vancomycin, teicoplanin, and bacitracin mainly treat Gram-positive bacteria. However, in our study, these three peptide antibiotics combined with rifampicin had 100% additive effects. We proposed that rifampicin inhibited the β subunit of bacterial RNA polymerase, while these three peptide antibiotics disrupted the bacterial cell wall synthesis, these combinations may disturb cell reproduction in different steps to enhance the antibacterial effects (Douthit et al., 2020; Yu et al., 2020). Therefore, rifampicin could become an ideal choice for combination therapy with peptide antibiotics. Tigecycline is used as the last line of defense against XDRAB. Our study depicted that combining five peptide antibiotics had different synergistic or additive effects on XDRAB. Notably, the synergistic and additive effects of tigecycline with polymyxins were 67% and 33%, respectively. Another study demonstrated that tigecycline at the sub-MIC level could reduce the biofilm formation of *A. baumannii* by controlling the expression levels of virulence factors connected with fimbriae and efflux pumps (Navidifar et al., 2019). Furthermore, the specific combination also showed a partially indifferent effect. For example, an indifferent effect occurred in 100% of XDRAB when vancomycin was combined with imipenem. However, the synergistic and additive effect of teicoplanin, a glycopeptide, in combination with imipenem, was 33% and 67%, respectively, similar to a previous study by Goldstein et al. (2004). Their study revealed that vancomycin had significant antagonism against glycopeptide intermediate *Staphylococcus aureus* infection (GISA) with β-lactam drugs, but teicoplanin helped enhance the antibacterial effect. The clinical implications for these findings are still unclear. Due to the limitations of the *in vitro* activity, further studies through *in vivo* models should be conducted to investigate the mechanism of action involving peptide antibiotics with other antimicrobials to combat *A. baumannii* associated infections.

## Conclusion

Our study has revealed that five clinically available peptide antibiotics combined with five antimicrobials produce different antibacterial effects on nine XDRAB strains within planktonic and biofilm cells. The best combinations were polymyxins with imipenem and polymyxins with azithromycin, respectively, with 100% synergy against nine XDRAB strains in planktonic and biofilm cells. Furthermore, these combinations reduced the dose and enhanced the antibacterial effects against two forms of XDRAB, showing potential clinical significance against XDRAB infection.

## Data Availability

The original contributions presented in the study are included in the article/Supplementary Material, further inquiries can be directed to the corresponding author.
